# Characterization of Glycolysis-Associated Molecules in the Tumor Microenvironment Revealed by Pan-Cancer Tissues and Lung Cancer Single Cell Data

**DOI:** 10.3390/cancers12071788

**Published:** 2020-07-04

**Authors:** Jinfen Wei, Kaitang Huang, Zixi Chen, Meiling Hu, Yunmeng Bai, Shudai Lin, Hongli Du

**Affiliations:** School of Biology and Biological Engineering, South China University of Technology, Guangzhou 510006, China; 201810107408@mail.scut.edu.cn (J.W.); huangkaitang@foxmail.com (K.H.); bizic_chen@mail.scut.edu.cn (Z.C.); humeiling@scut.edu.cn (M.H.); 201820136048@mail.scut.edu.cn (Y.B.); linsd@scut.edu.cn (S.L.)

**Keywords:** glycolysis, tumor hypoxia microenvironment, pan-cancer, cancer single-cell, cell proliferation, P4HA1, HSPA8

## Abstract

Altered metabolism is a hallmark of cancer and glycolysis is one of the important factors promoting tumor development. There is however still a lack of molecular characterization glycolysis and comprehensive studies related to tumor glycolysis in the pan-cancer landscape. Here, we applied a gene expression signature to quantify glycolysis in 9229 tumors across 25 cancer types and 7875 human lung cancer single cells and verified the robustness of signature using defined glycolysis samples from previous studies. We classified tumors and cells into glycolysis score-high and -low groups, demonstrated their prognostic associations, and identified genome and transcriptome molecular features associated with glycolysis activity. We observed that glycolysis score-high tumors were associated with worse prognosis across cancer types. High glycolysis tumors exhibited specific driver genes altered by copy number aberrations (CNAs) in most cancer types. Tricarboxylic acid (TCA) cycle, DNA replication, tumor proliferation and other cancer hallmarks were more active in glycolysis-high tumors. Glycolysis signature was strongly correlated with hypoxia signature in all 25 cancer tissues (r > 0.7) and cancer single cells (r > 0.8). In addition, *HSPA8* and *P4HA1* were screened out as the potential modulating factors to glycolysis as their expression were highly correlated with glycolysis score and glycolysis genes, which enables future efforts for therapeutic options to block the glycolysis and control tumor progression. Our study provides a comprehensive molecular-level understanding of glycolysis with a large sample data and demonstrates the hypoxia pressure, growth signals, oncogene mutation and other potential signals could activate glycolysis, thereby to regulate cell cycle, energy material synthesis, cell proliferation and cancer progression.

## 1. Introduction

Altered metabolism is a hallmark of cancer [1,2]. Rapidly proliferating tumor cells consume glucose at a higher rate compared to normal cells and part of their glucose carbon is converted into lactate, this is referred to as the ‘aerobic glycolysis’ [3] which has been correlated with advanced tumor progression [4], treatment resistance [5,6] and poor clinical outcome [7,8] in various cancers. Numerous studies have confirmed that glycolysis intermediate can rapidly meet the energy requirement for cell proliferation and the material needs for fatty acids and nucleotides [9]. These studies have established that glycolysis can provide favorable conditions for tumor proliferation and thus plays a pivotal role in cancer development.

Previous studies have showed that glycolysis seems to be a consequence to oncogenic signaling activation such as P53 [10], MYC [11], MAPK [12] and PI3K-AKT signaling [13]. However, the role of the microenvironment in driving metabolism alteration is increasingly recognized in recent years [14,15]. Hypoxia is one of the key physiological and microenvironmental differences between tumor and normal tissues. It induces DNA amplification [15,16] and increases clonal selection [17], resulting in aggressive cancer phenotypes. A crucial results underlying such condition in a highly hypoxia tumor microenvironment (TME) is metabolic reprogramming of tumor cells [18], such as, glycolysis is an adaptation to this low oxygen pressure microenvironment for activation by hypoxia-inducible factor 1 subunit alpha (HIF1A) which can stimulate glycolytic by transactivation genes involved in *SLC2A1* and *ALDOA* [19]. These prior studies provide the interesting insights into the interplay between specific glycolysis genes with hypoxia environment or other molecular oncogenic signal. However, the main mechanisms driving the glycolysis remains unknown nor how glycolysis affecting tumor progression remains incompletely understood. Therefore, there is an urgent need for understanding the comprehensive adjustment manner of regulation of glycolysis. 

Although researchers have employed positron emission tomography (PET) following injection of the glucose analogue ^18^F-fluorodeoxyglucose (FDG) to diagnose tumour glycolytic ability and attempted to research the related mechanism [20,21,22]. However, not all cancers avidly take up FDG. Breast cancers, for example, show up to 20-fold differences in their FDG-PET signal that is attributed by histopathologic heterogeneity [23]. Besides, FDG-PET cannot possible easily applied to large cohorts of patient samples with large data volume. Thus, there is still a lack of molecular characterization of glycolysis and comprehensive study of molecules related to tumor glycolysis. To fill this gap, we defined the glycolysis level using a 22-gene expression signature and evaluated it in 9229 samples and 7875 single cells to create a pan-cancer quantification and explore glycolysis-associated molecular features in great depth. This is the first study to identify gene expression signatures that reflects glycolysis activity, which can be easily applied to large numbers of patient samples. We calculated the glycolysis distribution, compared the difference of clinical prognosis, genomic instability and discovered the correlation between hypoxia and glycolysis across multiple tumor types. We also confirmed these results with 7875 single cells from the representative human lung tumor [24]. Our study strongly suggests that glycolysis promotes tumor proliferation, is affected by various influencing factors. Furthermore, it provides the theoretical basis for understanding critical roles of metabolic alteration, which is the evolutionary choice to resist the pressure from the external environment and cancer cell proliferation in cancer progression and suggests a framework to guide therapeutic optimization to block the glycolysis and control tumor progression. 

## 2. Results

### 2.1. Classification of Tumor Glycolytic Activity by a Gene Expression Signature

**Figure 1:**
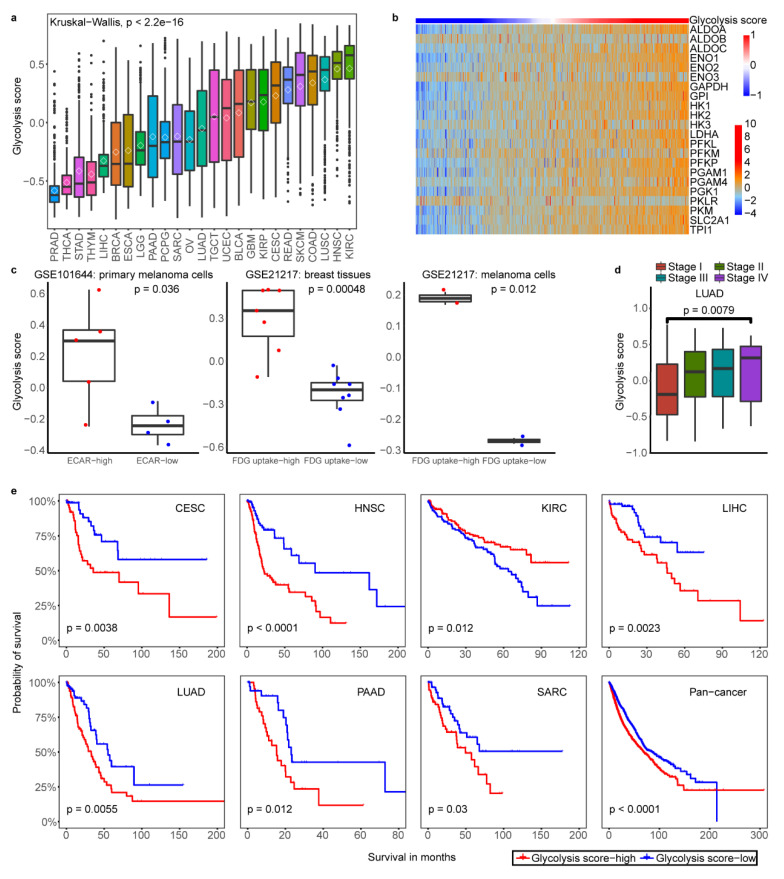
Validation of a 22-gene expression signature for glycolysis activity and clinical significance of glycolysis. (**a**). Glycolysis scores based on the mRNA abundance signature in 25 tumor types, sorted by the median GSVA score (horizontal black line) and mean score (diamond pattern) for each cancer type. (**b**). Samples are ordered from lowest to highest glycolysis score with 22-gene expression (z score of log_2_(TPM+1)) distribution in UCEC. The top color bar shows glycolysis score. (**c**). Glycolysis scores of cancer cell lines and tumor fragments under FDG high uptake and low as well as ECAR high and low conditions in three datasets. A two-sided Student’s T-test was used to assess the difference. *p*  <  0.05. (**d**). The glycolysis score was higher in clinical stage IV compared with stage I in LUAD, T-test was used to assess the difference. *p*  <  0.05. (**e**). Kaplan–Meier curves show that glycolysis score-high status is associated with worse survival time in multiple cancer types. A two-sided log-rank test *p*  <  0.05 is considered as a statistically significant difference.

To classify the glycolysis activity of tumor samples, we choose a 22-gene expression signature to represent the glycolytic activity (see Methods). The samples were classified according to glycolysis score by gene set variation (GSVA) analysis across all cancer types (Figure 1a) and in each cancer type (Table S1), respectively. The proportions of glycolysis score greatly varied among different cancer types, with some cancers having high glycolysis such as kidney renal clear cell carcinoma (KIRC), head and neck squamous cell carcinoma (HNSC), lung squamous cell carcinoma (LUSC), and colon adenocarcinoma (COAD) while some other cancer types having low glycolysis such as prostate adenocarcinoma (PRAD), thyroid carcinoma (THCA), stomach adenocarcinoma (STAD), and thymoma (THYM) (Figure 1a). Compared with normal tissues, glycolysis score was significant higher in tumors across cancer types except liver hepatocellular carcinoma (LIHC), STAD and PRAD (Figure S1a). In the 25 cancer types and 12 corresponding normal tissues surveyed, glycolysis score-high groups (the samples with top 30% of glycolysis scores in each cancer types), -low groups (the samples with bottom 30% of glycolysis scores in each cancer types) and normal groups all contained ≥ 30 samples in per each cancer type (Table S1). To examine whether glycolysis score was driven by modest differences in the levels of many members or more dramatic effects on only 1 to 2 key enzymes, we determined the contribution of individual enzymes to overall pathway enrichment based on 22-gene expression in all tumor samples. Genes encoding enzymes showed the most consistent enrichment within the glycolysis score in pan-cancer (Figure S1b) and also in one of the cancer types uterine corpus endometrial carcinoma (UCEC) with the large sample size more than 500 (Figure 1b). We performed analyses to validate robustness of gene signature using the GEO data in three independent gene expression datasets of cancer cell lines and tumor fragments with high and low glycolysis capacity determined by PET using ^18^F-FDG uptake or characterized by extracellular acidification rate (ECAR). We found a strong association between measured FDG uptake or ECAR and glycolysis score (Figure 1c). The observed consistency suggests that based on 22-gene signature is appropriate to represent glycolysis activity in different cancer types.

To examine whether tumors with the high and low glycolysis were clinically distinct, we next determined glycolysis activity whether having relations with tumor stage and patient overall survival. The higher glycolysis activity was associated with tumor advanced-stage in lung adenocarcinoma (LUAD) and breast invasive carcinoma (BRCA) (Figure 1d, Figure S1c). We observed that glycolysis score-high tumors were consistently associated with lower overall survival in several cancer types in Kaplan Meier survival analysis with log-rank test, such as pancreatic adenocarcinoma (PAAD), LUAD and pan-cancer pattern (*p* < 0.05)(Figure 1e). The multivariate survival analysis showed that glycolysis score and hypoxia score were considered as the same variable factors in some cancer types which indicated glycolysis score was not an independent prognostic factor in a pan-cancer landscape (Table S2). These results highlighted the clinical relevance of metabolic subtypes in some cancer types and suggested the potential prognostic power of glycolysis activity classification.

### 2.2. The Genomic Alteration in Glycolytic Score High Tumors

**Figure 2:**
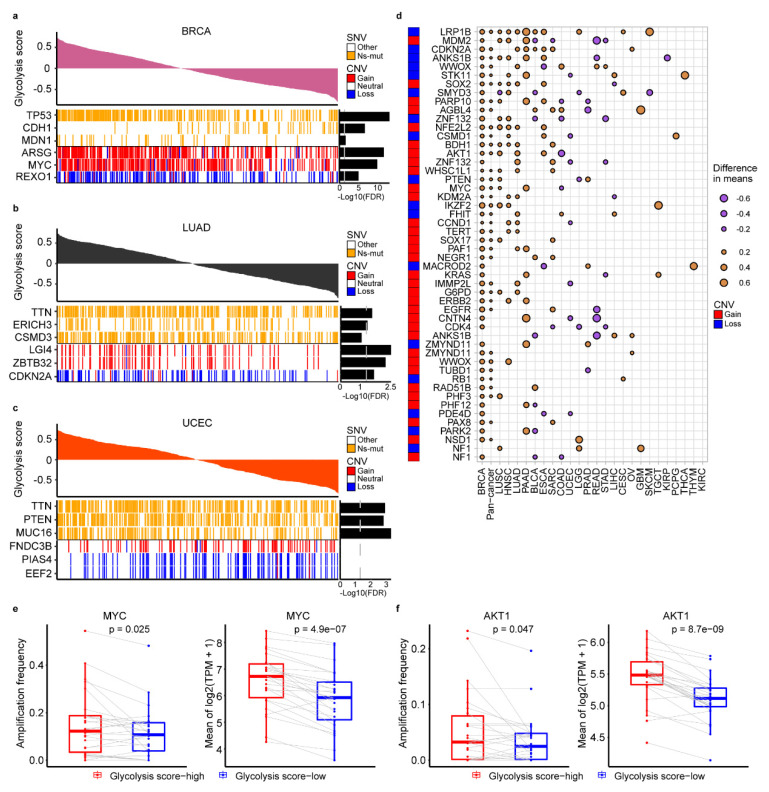
Characteristics of genomic changes associated with glycolysis activity. (**a**–**c**). Notable associations of somatic single-nucleotide variants (SNVs) and copy number aberrations (CNAs) with tumor glycolysis in subjects with BRCA (**a**), LUAD (**b**) and UCEC (**c**). Benjamini and Hochberg adj.*p* values are on the right (T test). (**d**). Association of glycolysis with CNAs in oncogenes and tumor suppressor genes (T test). Dot size indicates the difference in mean glycolysis score between tumors with a CNA (gain for oncogene, loss for tumor suppressor gene) and those without a CNA. (**e**). Amplification frequency of *MYC* (left) and mean expression of *MYC* (right) is higher in glycolysis-high than low tumors across 25 cancer types. (**f**). Amplification frequency of *AKT1* (left) and mean expression of *AKT1* (right) is higher in glycolysis-high than low tumors across 25 cancer types. A paired Student’s t-test *p*  <  0.05 is considered as a statistically significant difference.

We next sought to identify genomic changes that characterize tumor glycolysis. To assess whether glycolysis was associated with an elevated CNVs rate and somatic single-nucleotide variants (SNVs) features, we analyzed the difference of glycolysis score in samples with different CNVs or SNVs features across 25 cancer types (Tables S3 and S4) and focused on three tumor types with large sample more than 500 (BRCA, LUAD UCEC ) (Figure 2a–c). In BRCA, high glycolysis tumors were more likely to harbor loss of *REXO1* (adj. *p* < 10^-5^) and gain of *MYC* and *ARSG* (adj. *p* < 10^-10^) (Figure 2a). High glycolysis breast tumors also showed an elevated rate of *TP53* point mutations (adj. *p* < 10^-10^) and reduced *CDH1* mutations (adj. *p* < 10^-5^) (Figure 2a). Gain of *LGI4* and *ZBTB32* and loss of *CDKN2A* were observed in LUAD with high glycolysis (adj. *p* < 10^-2^) (Figure 2b). High glycolysis tumors in UCEC were associated with *MUC16*, *PTEN* and *TTN* mutations (adj. *p* < 10^-2^) (Figure 2c). Besides, alterations in other gene mutations were also associated with glycolysis in other cancer types, for example, higher *PIK3CA* mutation event was found in glycolysis-high group of COAD (adj. *p* = 10^-2^); *KRAS* and *TP53* mutation was found in glycolysis-high group of PAAD (adj. *p* < 10^-4^, adj. *p* = 10^-2^, respectively); *TTN* mutation was found in high glycolysis group of STAD (adj. *p* = 10^-2^) (Table S3). High glycolysis tumors were more likely to harbor loss of *RBL1* in COAD (adj. *p* < 10^-2^), loss of *MACROD2* in esophageal carcinoma (ESCA) (adj. *p* < 10^-2^), loss of *AOX1* in HNSC, loss of *ERBB4* in LUSC (adj. *p* < 10^-5^), loss of *PTEN* in PRAD (adj. *p* < 10^-6^) and gain of *CCND1* in HNSC (adj. *p* < 10^-2^) (Table S4).

Next, analysis of 114 cancer driver genes altered by CNAs [25] identified multiple oncogenes and tumor suppressors were associated with glycolysis activity in 25 cancer types (Figure 2d, Table S5). As showed in Figure 2d, loss of the tumor-suppressor gene *LRP1B* was associated with elevated glycolysis in 11 separate tumor types, whereas gain of the *AKT1* oncogene was associated with elevated glycolysis in five tumor types. In addition to BRCA, gain of *MYC* was also observed in PAAD and LUSC (*p* < 10^-2^). To explore how CNVs may influence these genes mRNA abundance, we compared the expression of these genes between glycolysis-high and -low tumors. We observed *MYC* and *AKT1* mRNA expression was consistent with copy number amplification across cancer types (Figure 2e–f, Figure S2a). Besides, correlation analysis showed *MYC*, *AKT1* mRNA expression and glycolysis score were positive correlated across a variety of cancers ranging from r = 0.10 to 0.57 for *MYC* and r = 0.15 to 0.50 for *AKT1* (*p* < 0.05) (Figure S2b). In addition, tumor mutation load burden (TMB) and tumor ploidy were also associated with glycolysis activity in our analysis. High glycolysis samples had significantly increased TMB in 11 cancer types, such as BRCA, STAD and UCEC (Figure S2c). The aneuploidy score in each tumor samples (Table S5), obtained from previous study [26], was positively or negatively correlated with glycolysis scores depending on specific cancer types, such as, in PAAD with r = 0.43 and rectum adenocarcinoma (READ) with r = -0.32 (*p* ≤ 0,05) (Figure S3a, Table S6). However, the glycolysis scores have no significant differences among samples without, with one and with two genome doubling (Figure S3b).

### 2.3. The Key Cancer Hallmarks and Metabolic Pathways in Glycolytic Score High Tumors

**Figure 3:**
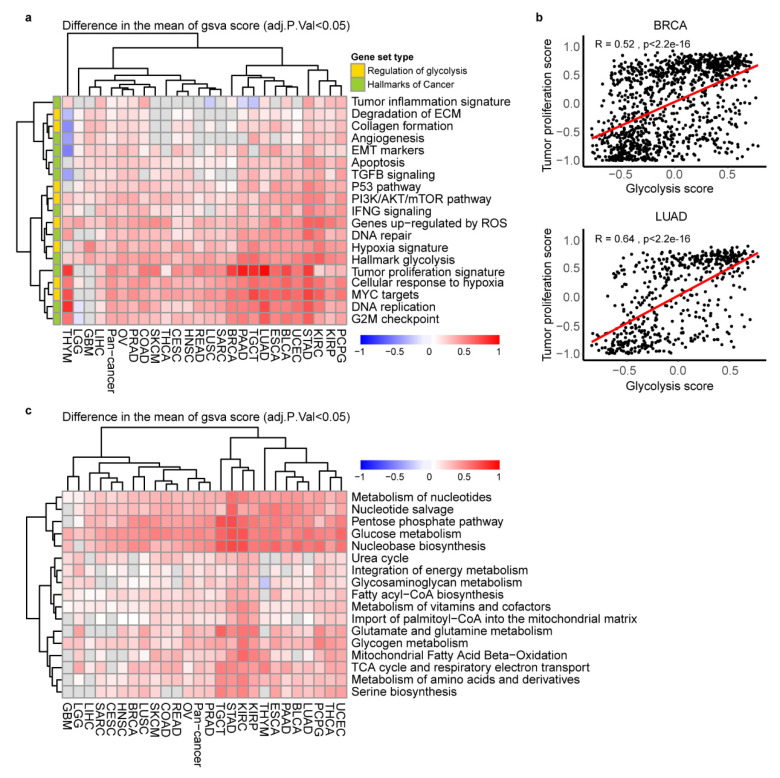
Association of glycolysis score with cancer hallmarks and metabolic reprogramming. (**a**). Heatmap showing the difference of GSVA scores of cancer hallmarks signatures enriched in the high glycolysis versus low tumors. adj.*p* < 0.05. (**b**). The Spearman correlation between tumor proliferation signatures and glycolysis score in BRCA and LUAD, *p*  <  0.05. (**c**). Heatmap showing the difference of gene set variation (GSVA) scores of cancer metabolic reprogramming signatures enriched in the high glycolysis versus low tumors. adj.*p* < 0.05.

To explore the differences in metabolic characteristics and cancer hallmarks of the glycolysis-high and -low groups in each cancer type (Table S7), we did differential signature enrichment based on GSVA analysis, using publicly available gene set in Molecular Signatures Database (MSigDB) database. Using independently gene signatures, we sought after differential enrichment in the glycolysis-high and low tumors (adj.*p* < 0.05 difference between the absolute means of GSVA scores in the two groups). The heat map combined with GSVA score difference showed that hallmark glycolysis, tumor proliferation signature, DNA replication, G2M checkpoint and MYC targets gene signatures were more active in high glycolysis groups across 23~24 cancer types (Figure 3a). As expected, tumor cell proliferation signature score was positively correlated with glycolysis score in 23 cancer types (r = 0.11~0.67) (Figure 3b, Table S6), implying that the high glycolysis tumor is characterized by a high proliferation rate. In addition, we observed a statistically significantly activation in hypoxia signature and cellular response to hypoxia signature in 24 cancer types (Figure 3a). In the metabolism pathways, TCA cycle significantly up regulated in glycolysis high groups in 20 cancer types. Besides, metabolism of nucleotides, nucleotide salvage, pentose phosphate pathway, glucose metabolism, nucleobase biosynthesis were more active in glycolysis high groups across 24~25 cancer types. Glutamate and glutamine metabolism, glycogen metabolism, mitochondrial fatty acid beta-oxidation and respiratory electron transport, metabolism of amino acids and derivatives and amino acids-serine biosynthesis were enriched in glycolysis-high groups across 19–20 cancer types except THYM and glioblastoma multiforme (GBM) (Figure 3c). 

### 2.4. The Association between Glycolysis and Hypoxia

**Figure 4:**
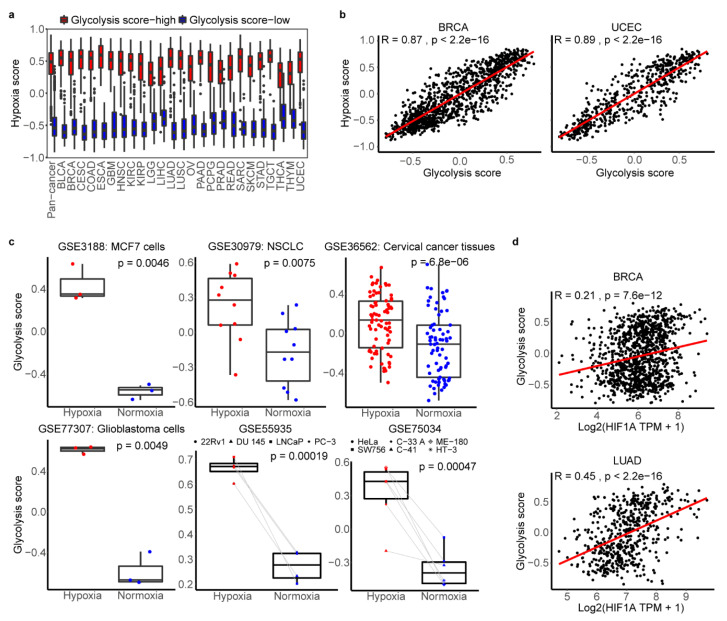
Association of glycolysis and hypoxia microenvironment. (**a**). The distribution of hypoxia score in glycolysis high and low tumors across cancer types. (**b**). Spearman correlation between tumor glycolysis score and hypoxia score in BRCA and UCEC. *p* < 0.05. (**c**). The distribution of glycolysis score of cancer cell lines and tumor fragments under hypoxic and normoxic conditions in six datasets. Two-sided Student’s t-test and paired Student’s t-test were used to assess the difference. *p* < 0.05. (**d**). Spearman correlation between tumor glycolysis score and *HIF1A* expression in BRCA and LUAD. *p* < 0.05.

Based on hypoxia signatures was significantly active in glycolysis-high tumors shown in the above result (Figure 3a), we sought to determine whether there is a relationship between them through the big data research. The hypoxia status was also defined by an established gene expression signature using GSVA, which was widely applied in previous researches [27,28,29]. Comparing with normal tissues, hypoxia score was significant higher in tumors across cancer types except kidney renal papillary cell carcinoma (KIRP), STAD and PRAD (Figure S4a). We compared hypoxia score in our glycolysis-high and -low groups found that hypoxia score was significantly higher in glycolysis-high tumors than the low one (Figure 4a, *p* < 2e-16). Next, by the calculation of spearman correlation, we found that glycolysis and hypoxia score were highly correlated with r > 0.7 across 25 cancer types (Figure 4b, Table S6). We compared the hypoxia scores in FDG-high vs. FDG-low and ECAR-high vs. ECAR-low datasets and found there was significant difference between two groups of breast cancer tissues in GSE21217 dataset with a large sample, which demonstrated the hypoxia score could distinguish samples of determined glycolysis levels to some extent (Figure S4b). We then compared hallmark hypoxia from mSigDb in our glycolysis high and low groups, and found that hallmark hypoxia score was significantly higher in glycolysis-high tumors than the low ones (Figure S4c). The hallmark glycolysis and hallmark hypoxia were also highly correlated with each other, and the correlation coefficient rang form 0.47 to 0.87 across cancer types, which suggested that high correlation between hypoxia and glycolysis is credible (Figure S4d, Table S6). To exclude the possibility that the overlapped genes (*LDHA*, *TPI1*, *ALDOA*, *ENO1*, *PGAM1* and *SLC2A1*) in both hypoxia and glycolysis gene sets contributed the high correlation, we calculated the correlation between glycolysis score (without overlapping genes, 16 genes) and hypoxia score. The results showed that they were highly correlated with each other (r = 0.48 ~ 0.9, *p* < 0.05) across 25 cancer types (Figure S4e, Table S6). To further exclude the possibility that the correlation was driven by a few correlated genes with high variation in expression levels while most other genes were not correlated, we did further correlation calculation between each gene in glycolysis set and hypoxia score or gene in hypoxia set and glycolysis score. The results confirmed that most of individual genes defining glycolysis score or hypoxia score were also positively associated with two scores (Figure S5a,b). 

Here, we also found the positive correlation between single *HIF1A* expression and glycolysis score in 18 cancer types (r = 0.17 ~ 0.52, *p* < 0.05), such as r = 0.45 in LUAD (Figure 4d, Figure S5c). Using six independent samples from GEO database, further verification showed tumor cells and tissues under the hypoxic conditions harbored significantly higher scores including glycolysis score, hallmark glycolysis score and hallmark hypoxia score than those in normoxic state (*p*< 0.01) (Figure 4c, Figure S6a–b). All these results showed the strong correlations and associations between glycolysis and hypoxia.

### 2.5. Glycolysis-Associated Gene and Pathway across Cancer Types

**Figure 5:**
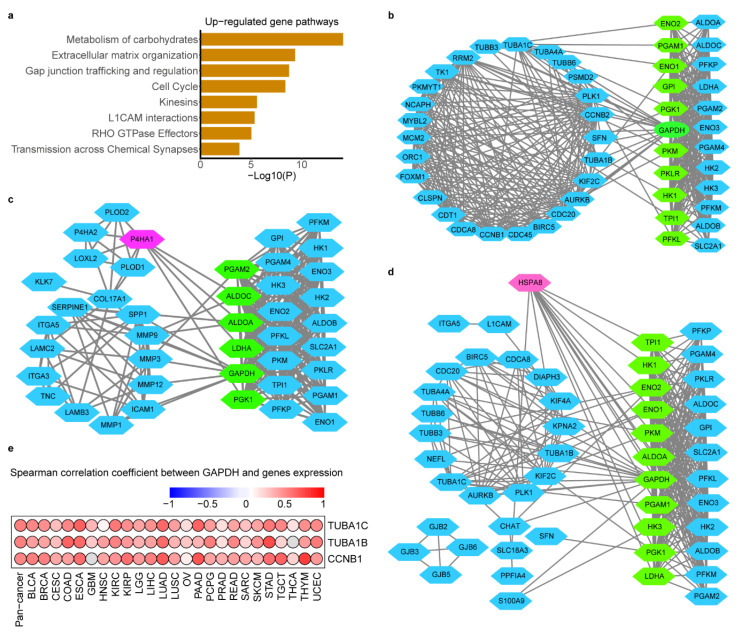
Glycolysis-associated mRNA and pathway signatures in hypoxia microenvironment. (**a**). Reactome pathway enrichment of genes that were up-regulated in the glycolysis high tumors in at least 13 cancers. *p* < 10^-2^. b-d. Protein interaction network between genes in cell cycle (**b**), ECM remodeling (**c**) and Gap junking (**d**) with glycolysis genes. Blue nodes in left represent DEGs and blue nodes in right represent the glycolysis genes, green nodes represent key genes in glycolysis which mostly linked with DEGs and red nodes represent the genes with most linking with glycolysis genes. (**e**). Spearman correlation between tumor *GADPH* and most glycolysis-related genes *TUBA1C*, *TUBA1B* and *CCNB1* in cell cycle across cancer types.

To address whether transcriptome features would differentiate between glycolysis-high and -low tumors, we compared the transcriptomes of the two groups by analysis of differentially expressed genes (DEGs) (1.5-fold difference, adj.*p* < 0.05) coupled with reactome term enrichment analysis (adj.*p* < 0.01) of DEGs across cancer types (Table S8). From the DEGs, there were 251 genes up regulated in glycolysis-high tumors versus -low tumors in at least 13 cancer types. Not surprisingly, glycolysis pathway highly enriched in our defined glycolysis-high groups (Table S8). The matrix remodeling, cell cycle and gap junction pathways also showed significant enrichment in the glycolysis-high tumors (Figure 5a). Besides, DEGs that expressed higher in glycolysis-low tumors were enriched in various pathways, such as, in ABC transporters in lipid homeostasis (Figure S7a). 

To identify genes most associated with glycolysis and explore interaction mechanism of these genes with glycolysis, we screened the genes according to the significance of their enrichment pathway. These up-regulated DEGs in three most significantly (adj.*p* < 10^-6^) enriched pathways (ECM remodeling, cell cycle and Gap junking) were regarded as candidate genes. The Spearman correlation between above candidate genes with glycolysis score was carried out to screen the most glycolysis-related genes. *TUBA1C* (r was range from 0.29 to 0.66), *TUBA1B* (0.21~0.65), *P4HA1* (0.25~0.74), *HSPA8* (0.17~0.60), *CCNB1* (0.22~0.62) as the top five genes were highly correlated with glycolysis score across cancers (Figure S7b, Table S9). 

To explore how glycolysis may influence these genes mRNA abundance or how glycolysis may be regulated by their signals. We constructed the protein interactions (PPI) of candidate genes with glycolysis genes to confirm the protein relationship between them. As Figure 5 showed there were many linking among them, *GADPH* was identified the glycolytic genes to hold the most interaction with candidate genes while candidate gene *HSPA8* and *P4HA1* were interacted with most glycolytic genes (Figure 5b–d). As we know glycolysis enzyme could act nuclear function to regulate transcription of gene acting in cell cycle, such as GADPH [30], so we carried out correlation analysis of mRNA expression between *GAPDH* and cell cycle-related genes *TUBA1C*, *TUBA1B*, *CCNB1* and the results showed that GAPDH was positively correlated with them in 24~25 cancer types (Figure 5e, *p* < 0.05). 

### 2.6. Association of Glycolysis and HSPA8, P4HA1 

**Figure 6:**
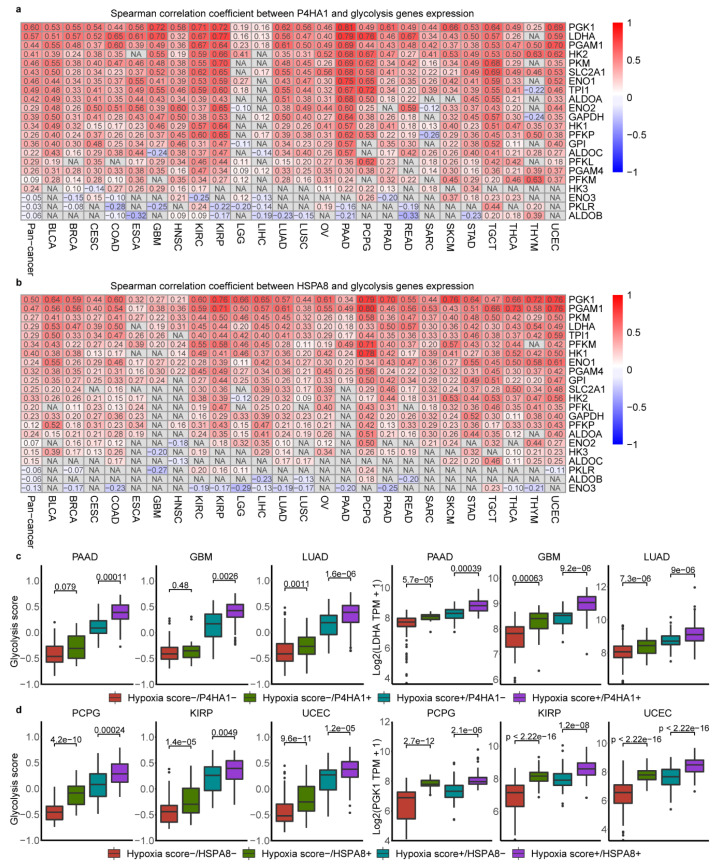
Association of glycolysis and P4HA1, HSPA8 in hypoxia microenvironment. (**a**). Spearman correlation of between *P4HA1* and 22 gene expression across cancer types. (**b**). Spearman correlation of between *HSPA8* and 22 gene expression across cancer types. (**c**). Glycolysis score levels and *LDHA* mRNA abundance differ depending on hypoxia status and *P4HA1* expression in three cancer types. Box plots represent the median (centerline) and upper and lower quartiles (box limits). Hypoxia score+ indicates tumors with top 50% hypoxia score; Hypoxia score− indicates tumors with bottom 50% hypoxia score. P4HA1+ indicates tumors with top 50% *P4HA1* expression in the Hypoxia score+ or Hypoxia score− groups, P4HA1− indicates tumors with bottom 50% *P4HA1* expression in the Hypoxia score+ or Hypoxia score− groups. (**d**). Glycolysis score levels and PGK1 mRNA abundance differ depending on hypoxia status and *HSPA8* expression in three cancer types. Hypoxia score+ indicates tumors with top 50% hypoxia score; Hypoxia score− indicates tumors with bottom 50% hypoxia score. HSPA8+ indicates tumors with top 50% *HSPA8* expression in the Hypoxia score+ or Hypoxia score− groups, HSPA8− indicates tumors with bottom 50% *HSPA8* expression in the Hypoxia score+ or Hypoxia score− groups.

As *HSPA8* and *P4HA1*, the top genes highly correlated with glycolysis score identified above, were also interacted with most glycolysis genes in the PPI analysis (Figure 5c–d), we hypothesized that HSPA8 and P4HA1 may be the potential factors influencing glycolysis. Thus, the correlation analysis of *HSPA8* and *P4HA1* with glycolytic genes was carried out. The results showed that *P4HA1* was correlated with *PGK1* (r = 0.16~0.81) in 25 cancer types and correlated with *LDHA* (r = 0.13~0.79) in 24 cancer types (*p* < 0.05) (Figure 6a). *HSPA8* was associated with most of glycolytic genes like *PGK1* (r = 0.21~0.79) and *PGAM1*(r = 0.17~0.80) across all cancer types (Figure 6b, *p* <  0.05).

To explore whether P4HA1 and HSPA8 affect glycolysis by regulation of glycolytic genes in hypoxia environment, we compared the differences of glycolytic score and key glycolytic gene expression at different hypoxia state and *P4HA1* or *HSPA8* expression patterns. As showed in Figure 6c, hypoxia with *P4HA1* mRNA abundance significantly predicted glycolysis score and *LDHA* expression across cancer types, such as, in GBM, LUAD and PAAD. The highest glycolysis score, *LDHA* and *PGK1* mRNA abundance were observed under high hypoxia and high *P4HA1* expression in some cancer types (Figure 6c, Figure S7c, Table S10). The same distribution as *P4HA1*, *HSPA8* was also proved to be a factor influencing glycolysis score and key glycolysis genes expression in different hypoxia state across cancer types. The highest glycolysis score, *PGK1* and *PGAM1* mRNA abundance were observed under high hypoxia and high *HSPA8* expression across cancer types (Figure 6d, Figure S7d, Table S10). The different expression of *HSPA8* and *P4HA1* was observed in cancer cell lines and tumor fragments of multiple cancer types under hypoxia compared with normoxic conditions from previous studies (GEO numbers see Methods). *P4HA1* was up regulated in cancer cells cultured with hypoxia across all single datasets (*p* <  0.05) (Figure S8a), however, *HSPA8* was showed the opposite trend in the same conditions in five datasets (*p* <  0.05) (Figure S8b). Collectively, our data show that *HSPA8* and *P4HA1* are strongly associated with glycolysis activity and correlated with key glycolytic genes such as *PGK1*. 

### 2.7. Landscape of Glycolysis at Single-Cell Level

**Figure 7:**
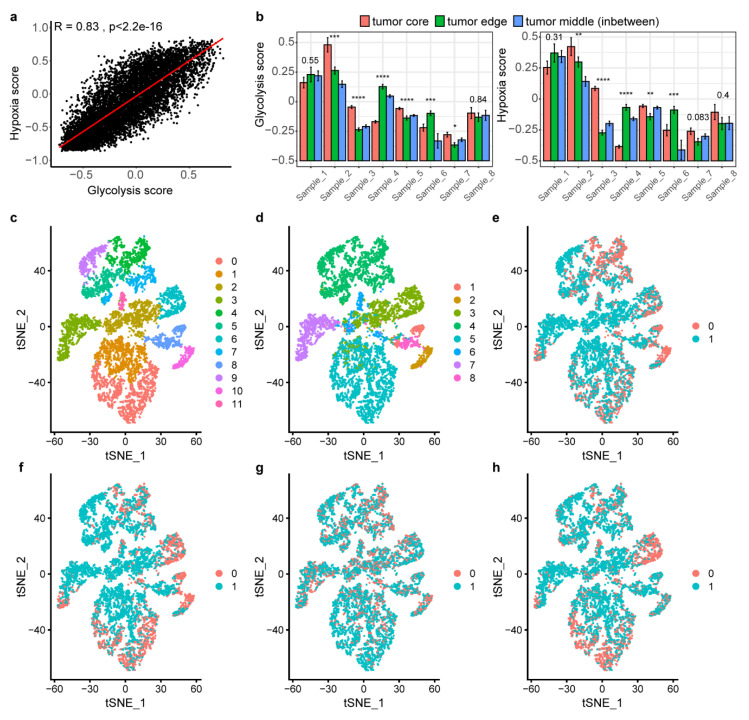
Landscape of glycolysis at lung cancer single-cell level. (**a**). The spearman correlation between hypoxia score and glycolysis score in single cells. (**b**). Glycolysis and hypoxia score size for each tumor-derived sample. The eight tumors were separated into three pieces, and each of them was marked as originating from either the core, the edge or the middle, in between edge and core. Bar plots indicate mean ± standard error of mean, * *p* < 0.05, ** *p* < 0.01, *** *p* < 0.001, **** *p* < 0.0001. (**c**–**h**). t-distributed stochastic neighbor embedding (t-SNE) of the tumor single cells profiled here, with each cell color-coded for (**c** to **h**): sub-clusters, the corresponding patient, the cells with top 30% of glycolysis score, the cells with top 30% of hypoxia score, the cells with top 30% of *HSPA8* expression, the cells with top 30% of *P4HA1* expression. Red-coded cells indicate top 30%, blue indicate the other 70% cells.

To further verify these glycolysis-related characteristics in single cells, we applied single-cell RNA-seq datasets for lung cancer [24] including an expansive set of gene expression of 7875 cells in 8 lung cancer patients. Using gene signature score, we classified the cells into glycolysis-high, -low group or hypoxia high, -low groups, separately. The Spearman correlation showed that glycolysis and hypoxia score was highly correlated with r = 0.83 (*p* <  0.05) in 7875 cells (Figure 7a), which was the very similar pattern observed in TCGA lung tumors (LUAD, r = 0.88; LUSC, r = 0.87, (*p* <  0.05)) (Table S6). We then assessed whether glycolysis-high cells reside more often in hypoxic areas of the tumor, using hypoxia and glycolysis score in each patients. As expected, high glycolysis always accompanied by hypoxia in cells no matter where cells divided from the tumor core, tumor edge or intermediate areas across 8 patients (Figure 7b). Intratumoral heterogeneity was observed that tumor core cells were observed in more hypoxic areas than the cells from the edge and in-between samples in four patients. 

To assess which genes differentially expressed between glycolysis-high and -low cells, we applied DEG analysis and found *P4HA1* was up regulated but *HSPA8* was not differentially expressed in glycolysis-high cells compared with -low cells (Table S8). Metabolism of carbohydrates including glycolysis was also the top enriched pathways in glycolysis-high cells (Table S8). Clustering these single cells revealed 11 clusters (Figure 7c). We attempted to identify similarity of cells between top 30% score of glycolysis (Figure 7e) and hypoxia (Figure 7f), top 30% expression of *HSPA8* (Figure 7g) and *P4HA1* (Figure 7h) in these clusters. We found that most cells with top 30% of hypoxia score, top 30% of glycolysis score, and top 30% expression of *P4HA1* are in the same site of each cluster (Figure 7e,f,h). This identified *P4HA1* and *HSPA8* as candidate factors underlying gene expression differences in glycolysis-high and -low tumor cells and in other TME stromal cells, respectively, seemed responsible for tumor-specific cell glycolysis phenotypes in hypoxia microenvironment.

## 3. Discussion

The underlying biology of the Warburg effect has remained obscure, such as, the biological events controlled by glycolysis pathway and the factors influencing glycolysis are not well defined. For this, our findings support the model that cancer cells favor glycolysis under hypoxia pressure, growth signals, as well as oncogene mutation, which is an important biological process that active cell cycle and promote cancer cell proliferation. Due to the lack of metabolomics data, it is necessary to elucidate mechanistic insights into the dysregulated metabolism of cancer cells utilizing transcriptome information. Based on a previous study, researchers use cancer patient cohort with parallel metabolite and transcriptomic profiling data to demonstrate that the metabolic gene expression indeed reflect metabolic activities [31], which provides confidence that this method is highly informative for study cancer metabolism. Besides, the metabolism-related studies have used mRNA expression patterns to reflect metabolic phenotype ranged from multiple cancer types to a single cancer type [32,33]. Here, we focus on the expression of metabolism-related genes to reflect the glycolysis and other metabolic activities. First, we applied a gene set including key coding glycolytic enzyme genes to represent glycolysis activity and verified this signature following a remarkably robust performance in validation cohorts. These genes have been verified by multiple studies [32,34] and database [35] for its importance in glycolysis. Second, congruent with previous studies [36], the glycolysis-high tumors were associated with a markedly worse prognosis than the -low tumors in some cancer types. Third, cell proliferation signals with synthesis of nucleic acid and other macromolecules were active in glycolytic-high tumors. Fourth, a strong positive correlation between the hypoxia and glycolysis activity was conserved in all cancer tissues and cancer single cells. Fifth, we confirmed that GADPH may active the cell cycle by activation the transcription of cycle-dependent protein. Finally yet importantly, based on the strong correlation between *HSPA8*, *P4HA1* and glycolysis score, glycolysis may be activated in hypoxic environment through other mechanisms not just HIF1A. 

Our analysis shows that the cell proliferation gene set is higher in glycolysis-high tumors relative to glycolysis-low tumors which is also mentioned by other studies [37]. As we know production of proteins, lipids and nucleic acids is essential for a successful replicative cell division and cell proliferation [38]. It has been shown that the biosynthesis of these highly needed macromolecules is achieved mainly through acquisition and utilization of sources of nutrients from its metabolic intermediates of glycolysis [38]. Our results show that glycolysis-high tumors harbor more active of these cellular biosynthetic pathways than low tumors, which can partly explained the way of promoting cell proliferation by glycolysis. However, we hypothesize that in addition to providing the materials and energy needed for cell growth, tumor glycolysis may activate other signals that promote cell proliferation, such as, by increasing cell cycle-dependent enzyme expression. The non-glycolytic functions of enzymes involved in glycolysis have recently been identified to be predominantly associated with the cancer development [39,40]. Cyclin B1 (CCNB1) is a regulatory protein involved in G2/M transition phase of the cell cycle and its encoding gene is highly correlated with glycolysis score and *GAPDH* expression across cancers. GAPDH, as the glyceraldehyde-3-phosphate dehydrogenase protein, catalyzes an important energy-yielding step in glycolysis metabolism. Various non-glycolytic functions of GAPDH have been reported in cancer, such as, it has been reported that GAPDH overexpression in cell nucleus is associated with cell cycle via its effect on cyclin B-cdk1 activity [30]. Up-regulation of CCNB1 is consistently associated with high-expressions of GAPDH in non-small-cell lung cancer [41]. Thus, these results support the hypothesis that GAPDH may affect *CCNB1* expression and cell cycle to promote cell proliferation in a pan-cancer landscape. However, this study has been limited to measuring transcriptional levels based on the RNA-seq data, and further experimentation is required to verify this phenomenon including cellular protein levels.

Several previous studies comparing gene expression between tumors and normal tissues have identified OXPHOS suppression as a recurrent metabolic phenotype in tumors [42,43]. In this study, we find that TCA cycle signature level is general higher in glycolysis-high tumors, which appears to support observations that tumor glycolysis can proceed with cellular mitochondria and in fact may be an adaptive response for tumor survival [44,45,46]. These observations indicate that TCA cycle are required for proliferation of most cancer cells and OXPHOS is not mutually exclusive with glycolysis as routes for energy production in adapting to the TME, which enables future efforts for therapeutic optimization to block both glycolysis and OXPHOS, thereby control tumor growth.

Despite a wealth of data linking glycolysis with oncogenes and tumor suppressor genes [47], most studies have focused on individual genes or in one special cancer type. We identify key modulators of glycolysis including the known factors and potential new ones uncovered by our analysis. It is well recognized that *AKT*, referred to as protein kinase B, and *MYC*, as a nuclear phosphoprotein, are the most prevalent driving oncogenes in cancer and the potentially regulatory function affecting glycolysis [48,49]. In the present study, amplification in *MYC* and *AKT1* show a strong consistency with transcript levels and glycolysis-high tumors harbor increased *MYC* and *AKT1* expression, suggesting that *AKT1* and *MYC* activation contribute the glycolytic activation in some cancer type. Oncogenes including *KRAS*, *TP53*, *PIK3CA*, *TTN*, *CDH1* and *MUC16* may be considered glycolysis-associated utations, as they mutated in glycolysis-high tumors. Previous research has confirmed that *KRAS* [50], *TP53* [51], *PIK3CA* [52] and *CDH1* [53] mutations participate in regulation of glycolysis to affect cancer development. *TTN* and *MUC16* may the potential regulators of glycolysis and the further research is needed to verify their roles and specific mechanism in glycolysis. 

Hypoxia is the result of an imbalance between oxygen delivery and oxygen consumption and is associated with the malignant phenotype [54]. This study identifies glycolysis has such a strong correlation with hypoxia in both tissue and single-cell samples indicating that glycolysis tends to be active in tumors cells under the hypoxia TME. The single-cell results indicate that the levels of oxygen is highly variable from one area to another area within the same tumor and its heterogeneous may depend on the location and timing of samples biopsied [55] and also confirm the concordant relationship between hypoxia and glycolysis in highly dynamic TME. There are genes with overlapping functions in hypoxia and glycolysis gene signatures, leading the challenge to disentangle their signatures transcriptionally and to distinguish their individual related-molecular features, which is the limitation of this study. As hypoxia and glycolysis are the closely related biological processes and the glycolysis is recognized to be partly affected by signal molecules caused by hypoxia, some of the glycolysis-associated molecules in this study are closely related to hypoxia. The previous authoritative study on hypoxia shows that *TP53*, *TTN* and *CDH1* mutations as well as *MYC* amplification and *PTEN* deletion are associated with hypoxia [17], which is also observed in this study (Figure 2) and these molecular features may be the regulators to glycolysis and they may serve as an intermediate bridge linking hypoxia and upstream of glycolysis. As increased glycolysis is necessary in cancer cells with induced cell growth and proliferation [38], the various downstream effects of glycolysis is also revealed in this study, for instance, the cell proliferation-essential macromolecules metabolism as well as cell cycle phase are altered following the changes with glycolytic activity (Figure 3). However, the further research urgently needs to demonstrate the use of gene-expression signatures for glycolysis quantification and to verify its robustness and reveal more independent glycolysis-related results.

Perhaps the most important aspect of effect of hypoxia on glycolysis is determined by the HIF signaling pathway [56]. As the tumor hypoxia is a spatiotemporal variable during cancer development, HIF1α responds rapidly to hypoxia but also to re-oxygenation, making it quite unstable in the context of clinical sample collection [57], which may question the role of HIF1α as a marker to detect hypoxia in clinical material. However, as a master regulator of cellular and systemic homeostatic response to hypoxia by activating transcription of several genes, HIF1α could stimulate glycolytic energy production by transactivation genes involved in extracellular glucose import (such as *SLC2A1* [58]) and can channel glucose into glycolysis by increasing the enzymes involved in this process (*TPI1*, *HK2*, *PFK1*, *ALDOA*, *ENO1* and *LDHA*) [19,59]. Our analysis of large transcriptomic profiles in a broader context clarifies that *HIF1A* expression correlated with glycolysis score and glycolysis is positively response to hypoxia in cancer cells. From the research so far, there are some glycolytic genes not regulated by HIF1α, we make assumptions that the glycolysis may be activated in hypoxic TME through other mechanisms not just HIF1A. Reactive oxygen species (ROS) levels, can be altered by hypoxia environment [60], also participates in regulating glycolysis through modulation of glycolytic enzymes, such as, post-translational modifications (PTMs) on enzymes. ROS-mediated direct oxidation of Cys (358) on PKM2 could decrease its activity and divert glucose flux into the pentose phosphate pathway and thereby allow cancer cells to withstand oxidative stress and support cell survival [61]. Therefore, ROS is a potential factors regulating glycolysis and is critical for cell proliferation and survival, which could expect the feasibility of combined approaches targeting ROS and metabolism for successful cancer therapy to become an exciting topic of study.

Given that HSPA8 is an chaperone by protection of the proteome from stress and the expression of *HSPA8* is highly expressed in tumor tissue [62], we assume it may play a critical role in mediating glycolysis coping with hypoxia pressure in tumor evolution. It shows that high glycolysis score is associated with increased *HSPA8* expression especially in hypoxia state, which point to it as a potential mechanism that may be responsible for glycolysis. In addition, *HSPA8* is up regulated in glycolysis-high tumors across cancer types but is no significant difference in cancer single cell. This incongruent phenomenon in tissues and single cell may be explained that cancer cells are coped with different pressure and duration in complex microenvironment. We assume that *HSPA8* may be expressed by other stromal cells and be an intermediate link responding to changing environmental pressures during the cancer evolution and activating the glycolysis reaction, and further study will be needed. Previously, the increased P4HA1 expression correlates with poor prognosis has been demonstrated in several cancers, including breast cancer, oral squamous cell carcinoma, melanoma, and prostate cancer [63,64,65,66]. However, as an active enzyme in collagen synthesis, we know little about how P4HA1 promotes tumor progression in the past. A recent study suggests that P4HA1 is essential for HIF-1 protein stability and is a new regulator of the HIF-1 pathway in breast cancer cells. The overexpression of *P4HA1* in cancer cells increases *LDHA* mRNA levels, and *P4HA1* expression correlates with *LDHA* mRNA levels in human breast cancer tissue [67], which are consistent with our finding. In summary, this study identifies P4HA1 and HSPA8 as the critical regulator in glycolysis and further work needed to verify our results and characterize their roles in activating glycolysis.

## 4. Materials and Methods 

### 4.1. Multi-omics Data and Clinical Data from TCGA, GEO and ArrayExpress

Level 3 molecular data, including mRNA expression, CNAs, SNAs and clinical data, including tumor stage, age, gender and overall survival times, across 25 cancer types were downloaded from the Cancer Genome Atlas (TCGA) data portal [68]. As TCGA focused on untreated primary tumors, the tissue samples for TCGA study were removed from the patient before the treatment [69]. The Gene Expression Omnibus (GEO) data was downloaded from NCBI GEO dataset [70] including GSE21217, GSE101644, GSE3188, GSE30979, GSE36562, GSE55935, GSE77307 and GSE75034 as validation data set. The signal cell RNA-seq data was downloaded from ArrayExpress under accessions E-MTAB-6149 and E-MTAB-6653.

### 4.2. Classification of Glycolysis Activity across Different Cancer Types

According to the reactome pathway database, gene expression and their importance in cancer, we selected a 22-gene expression signature (*SLC2A1*, *HK1*, *HK2*, *HK3*, *GPI*, *PFKL*, *PFKM*, *PFKP*, *ALDOA*, *ALDOB*, *ALDOC*, *TPI1*, *GAPDH*, *PGK1*, *PGAM1*, *PGAM4*, *ENO1*, *ENO2*, *ENO3*, *PKLR*, *PKM* and *LDHA*) that belongs the glycolysis core pathway [35]. To classify glycolysis status, we employed GSVA [71] to calculate the glycolysis score based on the 22-gene expression signatures (GSVA score was scaled from –1 to 1 in each sample). The glycolysis score was calculated across all cancer samples to compare the difference between cancers (Kruskal−Wallis test) and calculated in each cancer type to classify samples as glycolysis score-high and score-low groups using top and bottom 30% score samples, respectively. We included 25 cancer types in which both glycolysis score-high and low groups samples ≥ 30 for further analysis. The glycolysis score was compared in tumors and normal tissues in 12 cancer types with ≥ 30 normal samples (Wilcox rank test).

### 4.3. Clinical Relevance Analysis of Glycolysis Subtypes

We evaluated the associations of glycolysis score with two clinical features respectively: the patients’ overall survival time and tumor stage. The R package ‘‘survival’’ was used to perform the overall survival analysis and produce Kaplan-Meier survival plots. Univariate and multivariate Cox proportional hazard regression models were used to evaluate overall survival. HR and the 95% CI were generated using Cox proportional hazards models. As for associations of glycolysis score and tumor stage analysis, T test was performed to access the glycolysis score in different tumor stage.

### 4.4. Genomic Instability Associated with Glycolysis Activity

The association of glycolysis activity with CNAs and SNVs was tested in 25 cancer types and depicted in BRCA, LUAD and UCEC by using all samples with both CNA and SNV data (n BRCA = 1091, n LUAD = 513, n UCEC = 543). CNA and SNV biases were assessed for all genes for which data were available within these tumor types. The data of amplification, deletion and neutral status within a CNV threshold recorded as “1”, “−1” and “0”, respectively, was calculated by Gistic 2.0 [72]. CNA biases were tested for 19729 genes in each tumor types. The gain-loss (loss-gain) ratios for each gene were calculated based of the number of gain (loss) divided by the loss (gain) samples in each cancer type. One specific gene with gain-loss ratio > 2 would be defined gain or defined loss with loss-gain-loss > 2 in each cancer type [73]. Copy-number changes were associated with glycolysis activity by a comparison of glycolysis scores between tumors that were copy-number neutral to those with a copy-number gain [17] (or to those with a loss; T test). For each gene with SNV data, glycolysis scores were compared between tumors with a nonsynonymous mutation or without (T test). A Benjamini and Hochberg *p*-value adjustment was applied within each tumor-type cohort. Aneuploidy score and genome doubling of TCGA tumors were obtained from previous study [26] (Table S5) and the Spearman correlation was calculated between glycolysis score and aneuploidy score across cancer types. The glycolysis scores were compared among samples without, with one and with two genome doublings.

### 4.5. Pan-Cancer Associations of Driver CNAs and Glycolysis Activity 

Driver CNA associations were assessed within all 25 tumor types for previously described oncogenes and tumor-suppressor genes [25]. For each oncogene, differences in glycolysis scores were compared between tumors that were copy-number neutral and those with a copy-number gain. For each tumor-suppressor gene, differences in glycolysis scores were compared between tumors that were copy-number neutral and those with a copy-number loss (T test). The top 50 driver CNAs were plotted according to the number of tumor types in which each driver event had *p* < 0.05. 

### 4.6. Gene Set Enrichment

To calculate single-sample gene set enrichment, we used the GSVA program to derive the absolute enrichment scores of gene sets from several publications and previously experimentally validated gene signatures from MsigDB [74] as follows: tumor proliferation signature [27], tumor inflammation signature [75], cellular response to hypoxia, MYC targets, DNA replication, G2M checkpoint, P53 pathway, PI3K/AKT/mTOR pathway, IFNG signaling, genes up-regulated by ROS, DNA repair, degradation of ECM, collagen formation, angiogenesis, EMT markers, apoptosis, TGFB signaling, hallmark glycolysis, hypoxia signature and metabolism related pathways. To derive the GSVA score of each signature in each tumor sample, the normalized log2 (TPM+1) values were passed on as input for GSVA in the RNA-seq mode. Differentially enriched gene sets between the glycolysis high and low tumor groups were defined by GSVA adj.*p* < 0.05 (we used the limma R pachage because the GSVA scores were normally distributed around zero) [76].

### 4.7. Spearman Correlation of Glycolysis Score and Hypoxia Score

We selected a 14-gene expression signature (*ALDOA*, *MIF*, *TUBB6*, *P4HA1*, *SLC2A1*, *PGAM1*, *ENO1*, *LDHA*, *CDKN3*, *TPI1*, *NDRG1*, *VEGFA*, *ACOT7* and *ADM*) [27,28] that has been shown to perform the best when classifying hypoxia status. The GSVA was also employed to calculate the hypoxia score in each cancer type based on the gene signatures. The Spearman correlation was calculated between glycolysis score and hypoxia score across cancer types (*p* < 0.05). The hypoxia score was compared in tumors and normal tissues in 12 cancer types with ≥ 30 normal samples (Wilcox rank test).

### 4.8. Identification of Genes and Pathways Alterations between Glycolysis Score-High and Low Tumors

The difference of gene expression between glycolysis high and low groups was analyzed by edgeR (1.5-fold difference, adjust *p* < 0.05). We choose the genes co-upregulated in at least 13 cancer types as glycolysis positively related genes. Reactome pathway database [77] is an integrated database containing advanced functional information for the systematic analysis of gene functions, biological pathways and other research. Metascape [78] is an online resource to perform the gene annotation and functional enrichment analysis. We used Metascape to perform the pathway enrichment with the threshold of *p* value  <  0.01 and the number of enriched genes  ≥ 3 were concerned as significant. 

### 4.9. Data Processing in Single-Cell

The expression values were loaded into the Seurat package for the following analysis using the Seurat R package [79]. Cells that had either fewer than 200 RNA counts, below 200 or over 6000 expressed genes were excluded from the downstream analysis. Besides, representation of mitochondrial gene expression (e 10%) as a further quality control. The remaining 15,424 genes in 75,621 cells passed quailty control into the subsequent analysis. Sctransform function in Seurat was used to normalize. The list of highly variable genes were analyzed with principle component analysis (PCA) to conduct dimension reduction using RunPCA function. The significant PCA were then used to perform FindNeightbor and FindClusters function to get 10 clusters. FindAllmarker was used to identify the gene signatures of each cluster then to compare markers to Cellmarker database [80]. According to the known cell type markers, there were eight cell types defined including cancer cells, immune cells and other stromal cells. The 7875 cancer cells were extracted for subsequent analysis.

### 4.10. Subclustering of Single Cell Subtypes, Differential Gene Expression and Analysis of Molecular Feature Associated with Glycolysis 

The FindNeightbor and FindClusters function with resolution = 0.5 were used to cluster and get 11 subclsters. t-SNE method implemented in the Rtsne package with default parameters was used for visualization [24,81]. To assess differential genes and pathways between two sets of cells, we contrasted the gene expression for two groups using edgeR (1.5-fold difference, adjust *p* < 0.05).

Spearman correlation of glycolysis score and hypoxia score was performed as above in TCGA data. According to annotation file, tumor cells were from different samples divided into tumor core, tumor edge and intermediate. We used One-way analysis of variance (ANOVA) to compare the hypoxia and glycolysis score difference from different position in each patient. To visualize the distribution of cells characterized by hypoxia, glycolysis score, *HSPA8* and *P4HA1* expression, we color-coded for the top 30% score of hypoxia and glycolysis or top 30% expression of *HSPA8* and *P4HA1* in tSNE plot. 

## 5. Conclusions

Overall, our study offers a global landscape of glycolysis in human cancer cells from the highly complex TME. This work shows that the hypoxia pressure, growth signals, oncogene mutation and other potential signals could activate glycolysis, thereby to regulate cell cycle, energy material synthesis, cell proliferation and cancer progression. However, further work is needed to demonstrate the use of gene-expression signatures for glycolysis quantification and it will be necessary to validate our new findings in large independent cohorts, which will be the subject of future studies.

## Supplementary Materials

The following are available online at https://www.mdpi.com/2072-6694/12/7/1788/s1. Figure S1: 22-gene signature for glycolysis activity and clinical significance of glycolysis. Figure S2: Characteristics of CNAs and TMB associated with glycolysis activity. Figure S3: The association between glycolysis and tumor aneuploidy and genome doubling. Figure S4: The distribution of hypoxia score and the correlation with glycolysis across cancer types. Figure S5: Association between glycolysis and hypoxia across cancer types. Figure S6. Validation of gene expression signature for hypoxia status. Figure S7: Glycolysis-associated transcriptome signatures. Figure S8: Expression pattern of PAHA1 and HSPA8 in hypoxic and normoxic conditions. Table S1: TCGA cancer type descriptions and the clinical information. Table S2: Univariate and multivariate Cox regression analysis of the glycolysis score and survival of cancer patients in TCGA. Table S3: Glycolysis-associated SNAs in 25 cancers. Table S4: Glycolysis-associated CNAs in 25 cancers. Table S5: Pan-cancer regions of significant CNAs and aneuploidy score. Table S6. Correlation coefficient of glycolysis score and other signature score. Table S7: Signatures of cancer hallmarks. Table S8: DEGs and significantly enriched pathways in glycolysis-high versus glycolysis-low tumors and single cells. Table S9: The correlation between DEGs with glycolysis score across cancer types. Table S10: Association of glycolysis and P4HA1, HSPA8 in hypoxia microenvironment.

## Abbreviations

CNAs	Copy-number aberrations
TME	Tumor microenvironment
PET	Positron emission tomography
FDG	^18^F-fluorodeoxyglucose
ECAR	Extracellular acidification rate
GSVA	Gene set variation
t-SNE	t-Distributed stochastic neighbor embedding
SNVs	Somatic single-nucleotide variants
DEGs	Differentially expressed genes
PPI	Protein interactions
IHC	Immunohistochemistry
ROS	Reactive oxygen species
PTMs	Post-translational modifications
TCGA	The Cancer Genome Atlas
GEO	Gene Expression Omnibus
PCA	Principal component analysis
