# Supplementary material for: Characterization of Glycolysis-Associated Molecules in the Tumor Microenvironment Revealed by Pan-Cancer Tissues and Lung Cancer Single Cell Data
Source: Cancers (Basel). 2020 Jul 4;12(7):1788. doi: 10.3390/cancers12071788 (PMC7408567; doi:10.3390/cancers12071788)
Supplement: Supplementary file 1 [file cancers-12-01788-s001.zip › Supplementary Files/Supplementary Figures/SF8.pdf]

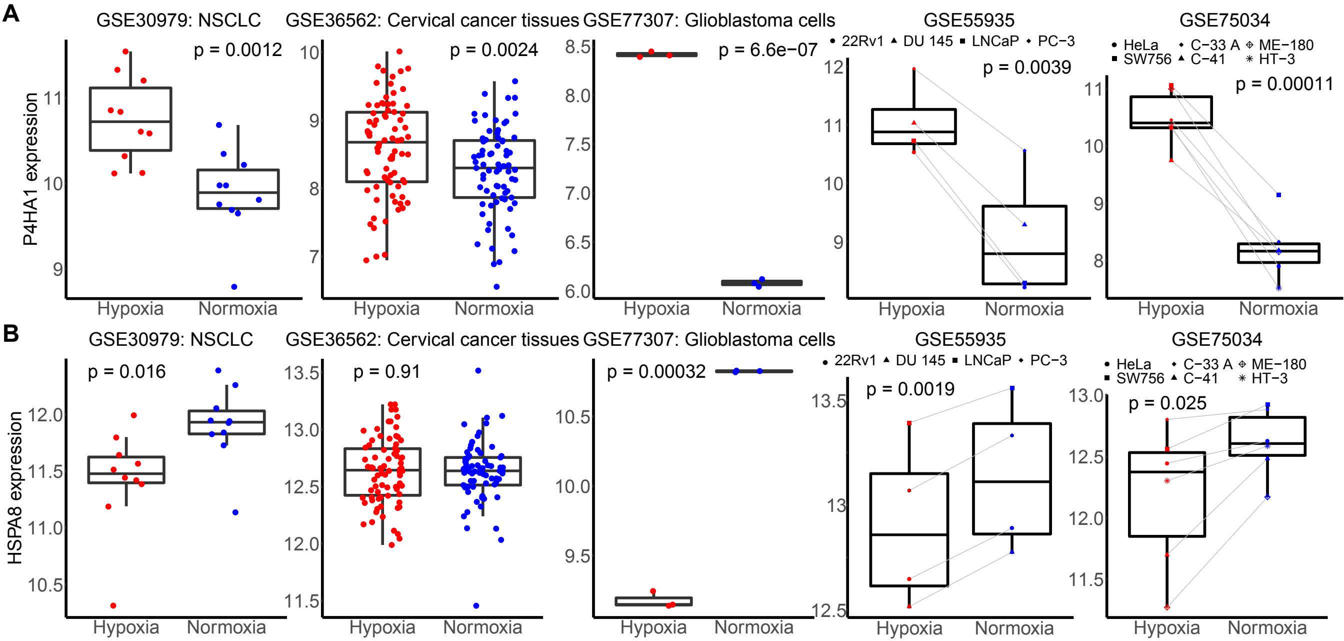

A
GSE30979: NSCLC
p = 0.0012
P4HA1 expression
Hypoxia
Normoxia
GSE36562: Cervical cancer tissues
p = 0.0024
GSE77307: Glioblastoma cells
p = 6.6e−07
GSE55935
22Rv1
DU 145
LNCaP
PC−3
p = 0.0039
GSE75034
HeLa
C−33 A
ME−180
SW756
C−41
HT−3
p = 0.00011
B
GSE30979: NSCLC
p = 0.016
HSPA8 expression
GSE36562: Cervical cancer tissues
p = 0.91
GSE77307: Glioblastoma cells
p = 0.00032
GSE55935
p = 0.0019
GSE75034
p = 0.025
