# Supplementary figures and images for: Characterization of Glycolysis-Associated Molecules in the Tumor Microenvironment Revealed by Pan-Cancer Tissues and Lung Cancer Single Cell Data
Source: Cancers (Basel). 2020 Jul 4;12(7):1788. doi: 10.3390/cancers12071788 (PMC7408567; doi:10.3390/cancers12071788)

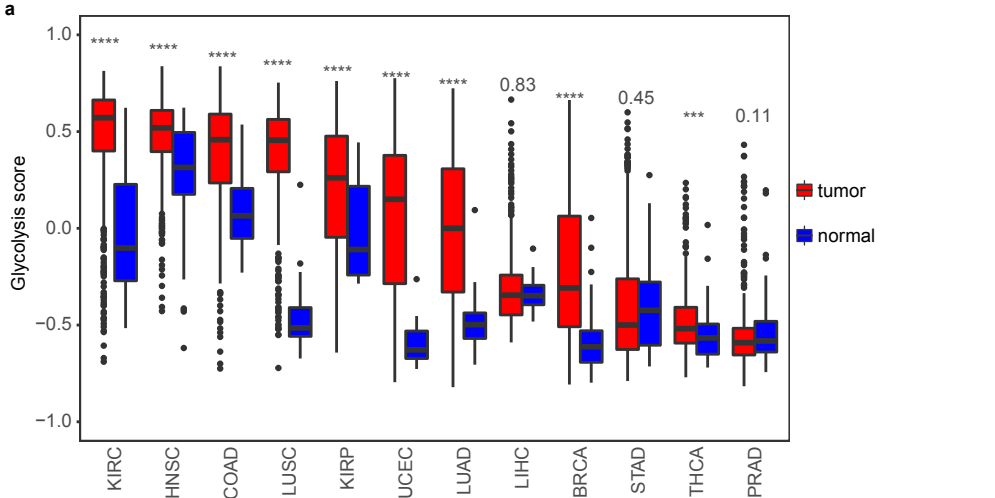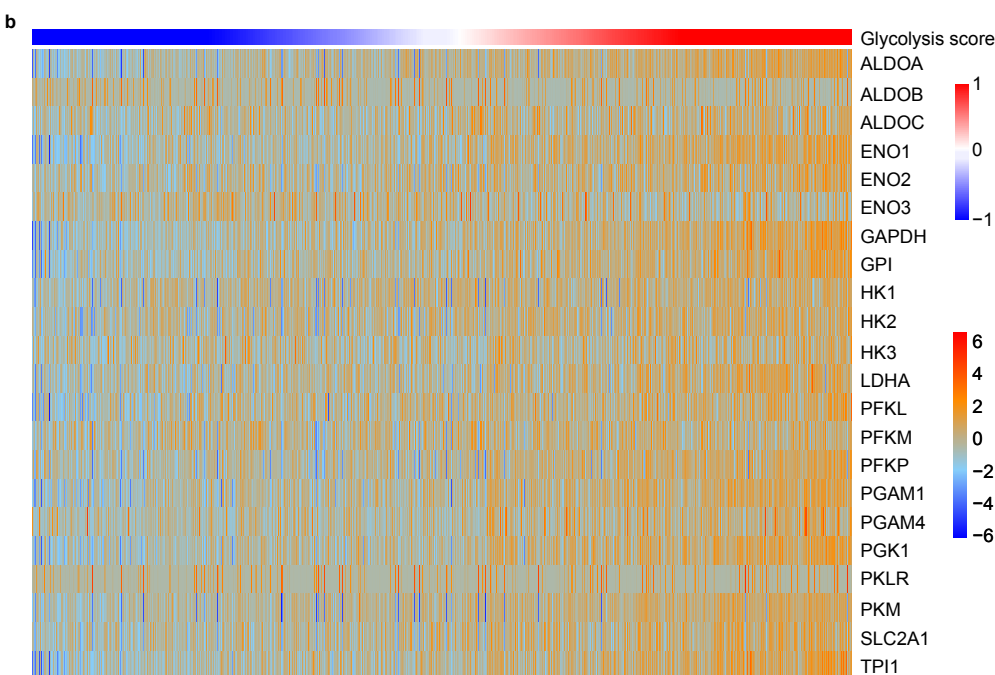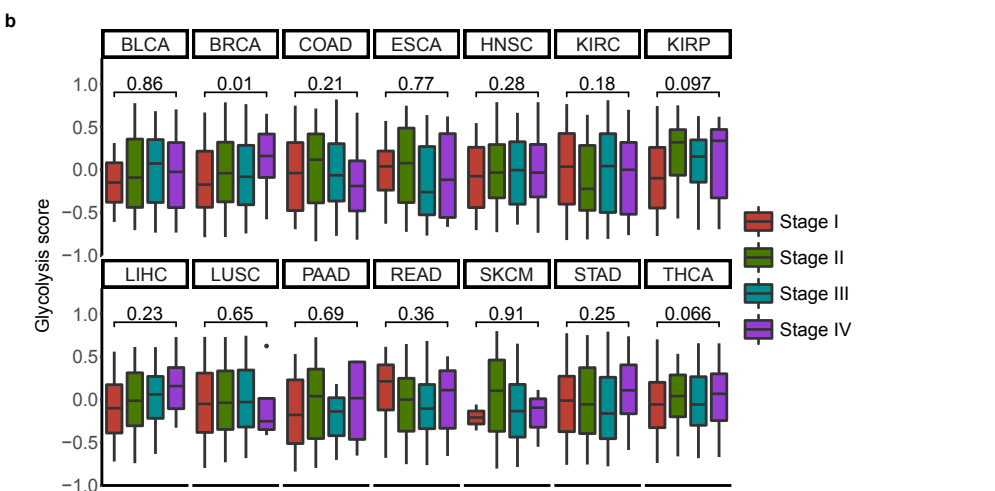

Supplement: Supplementary file 1 [file cancers-12-01788-s001.zip › Supplementary Files/Supplementary Figures/SF1.pdf]

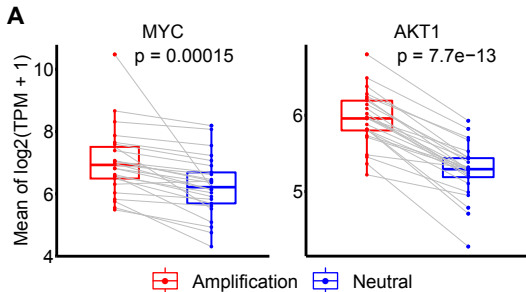

**C**

Glycolysis score-high Glycolysis score-low

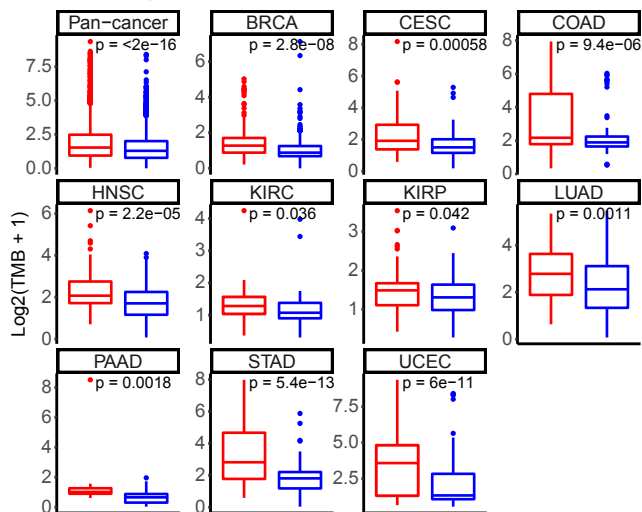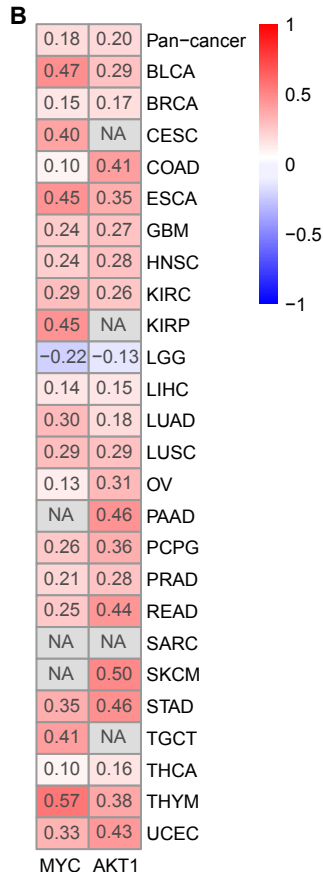

Supplement: Supplementary file 1 [file cancers-12-01788-s001.zip › Supplementary Files/Supplementary Figures/SF2.pdf]

a

Cancer type

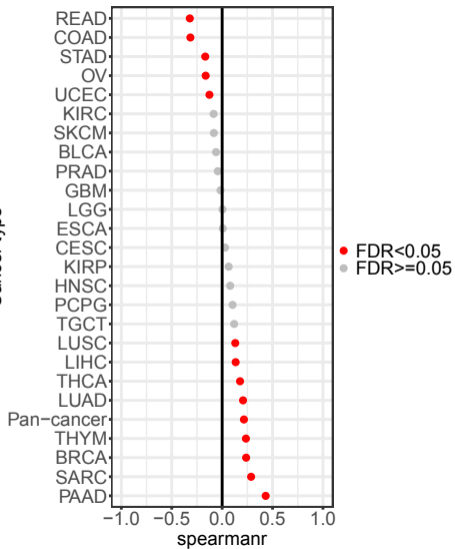

b

Glycolysis score

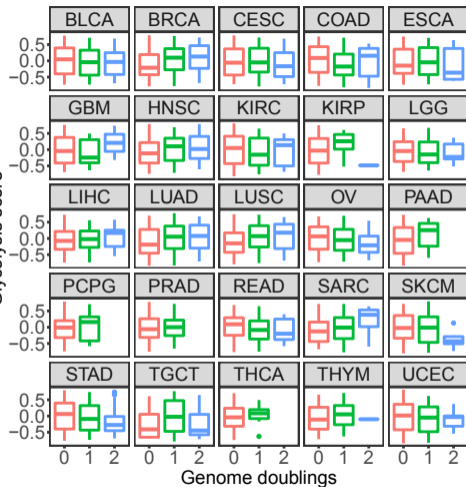

Supplement: Supplementary file 1 [file cancers-12-01788-s001.zip › Supplementary Files/Supplementary Figures/SF3.pdf]

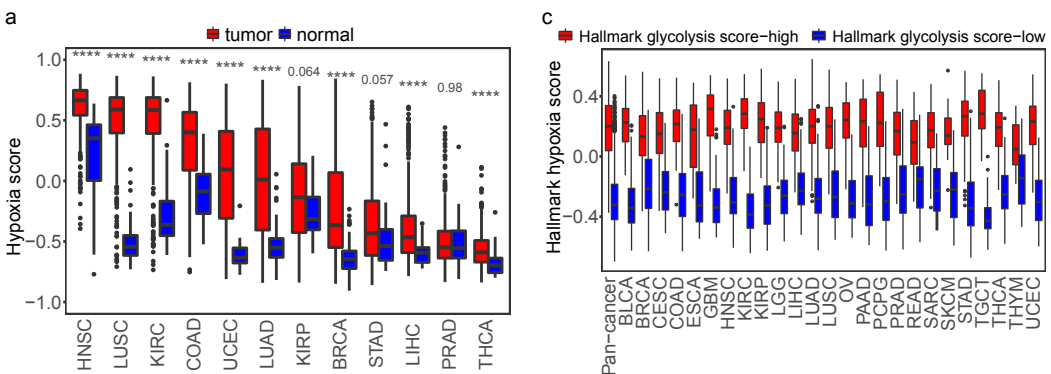

**b**

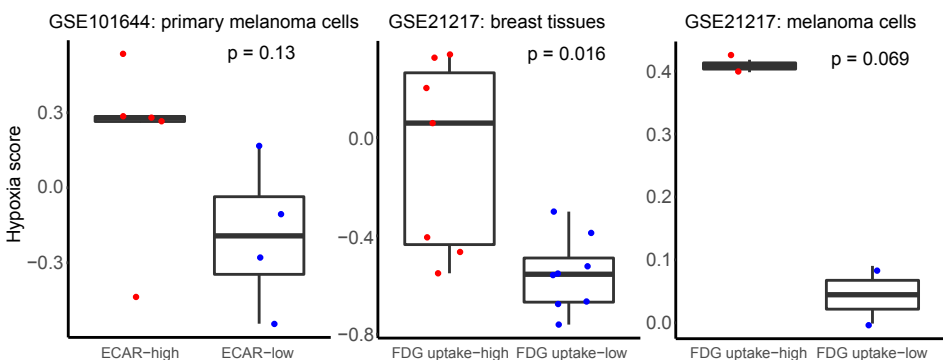

**d**

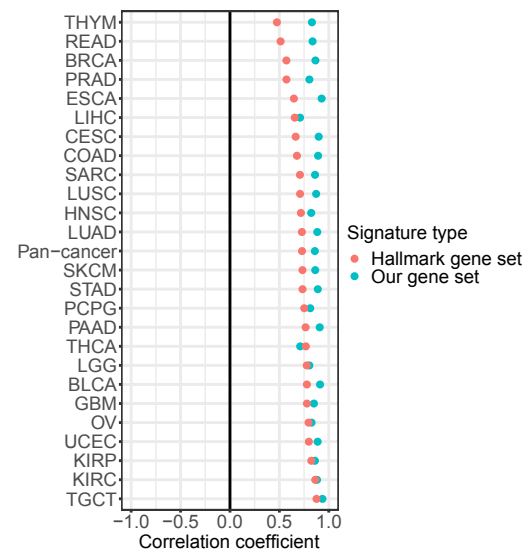

**e**

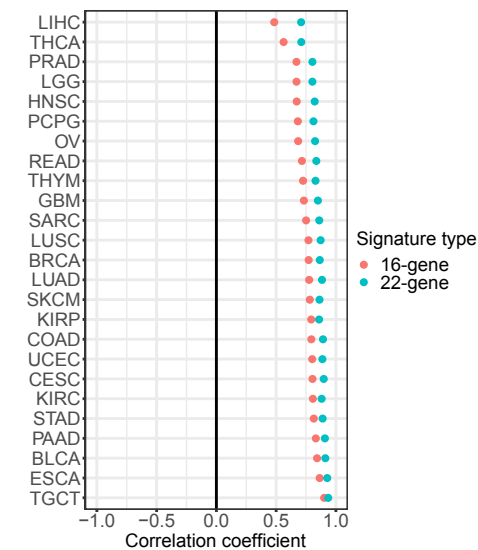

Supplement: Supplementary file 1 [file cancers-12-01788-s001.zip › Supplementary Files/Supplementary Figures/SF4.pdf]

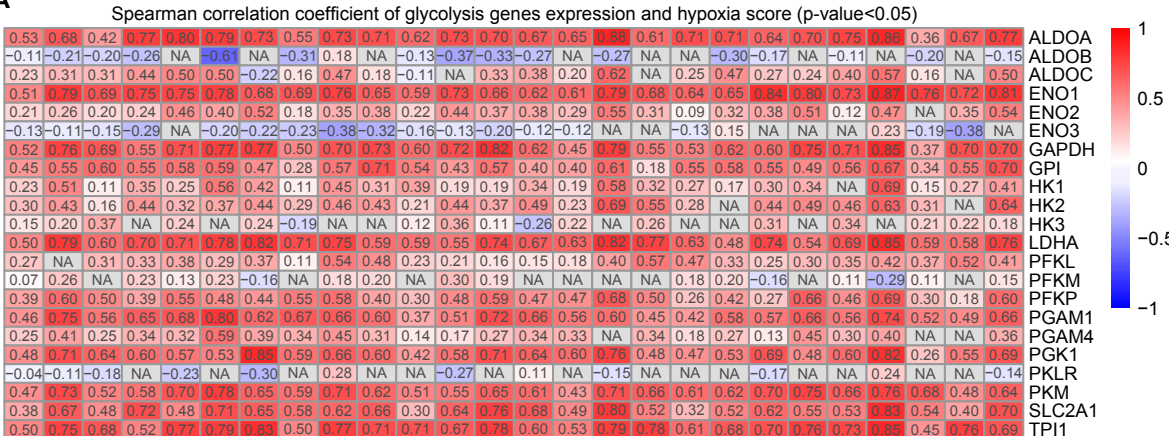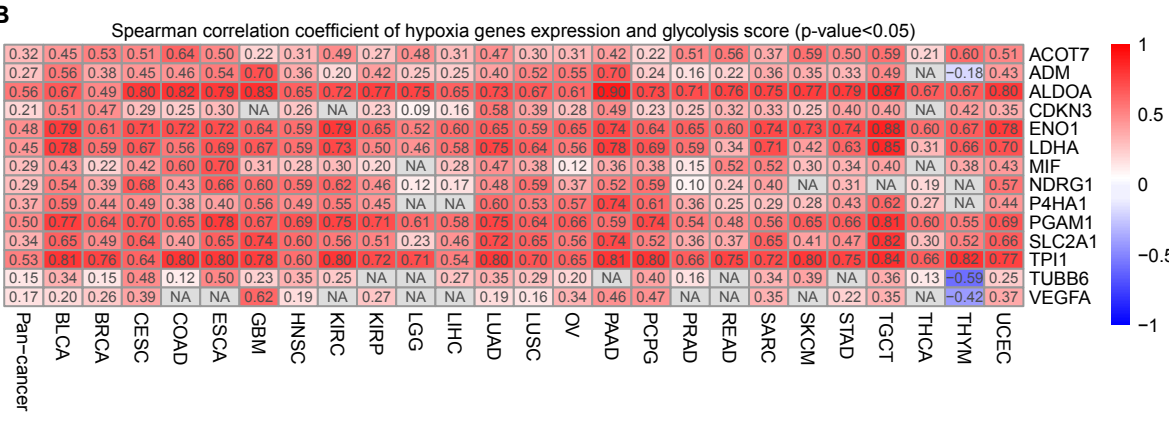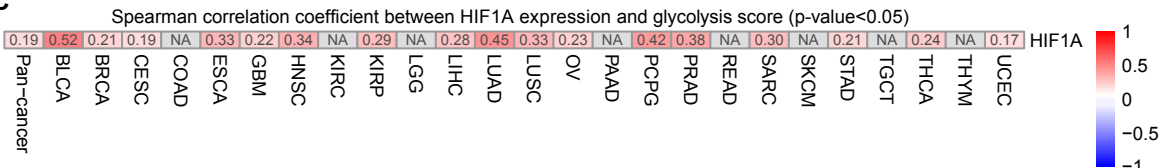

Supplement: Supplementary file 1 [file cancers-12-01788-s001.zip › Supplementary Files/Supplementary Figures/SF5.pdf]

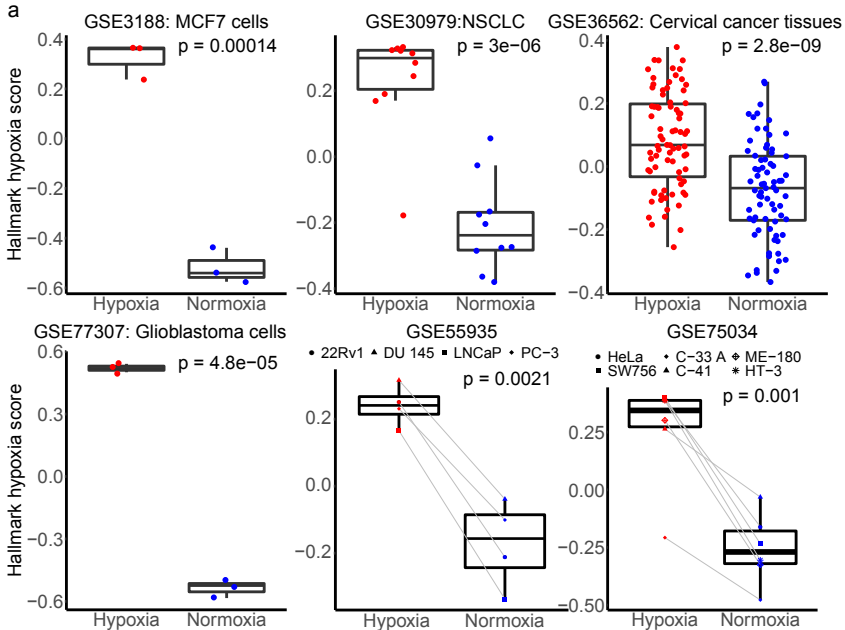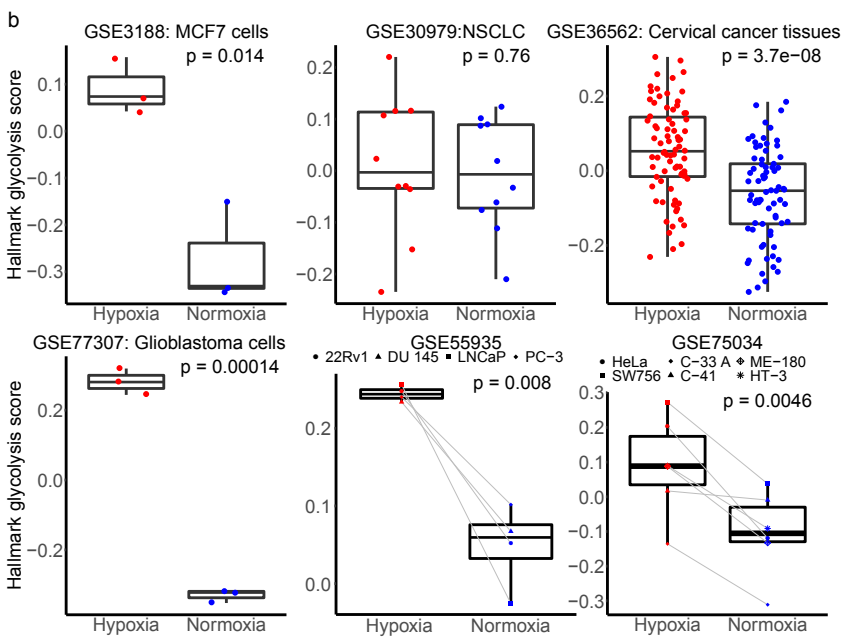

Supplement: Supplementary file 1 [file cancers-12-01788-s001.zip › Supplementary Files/Supplementary Figures/SF6.pdf]

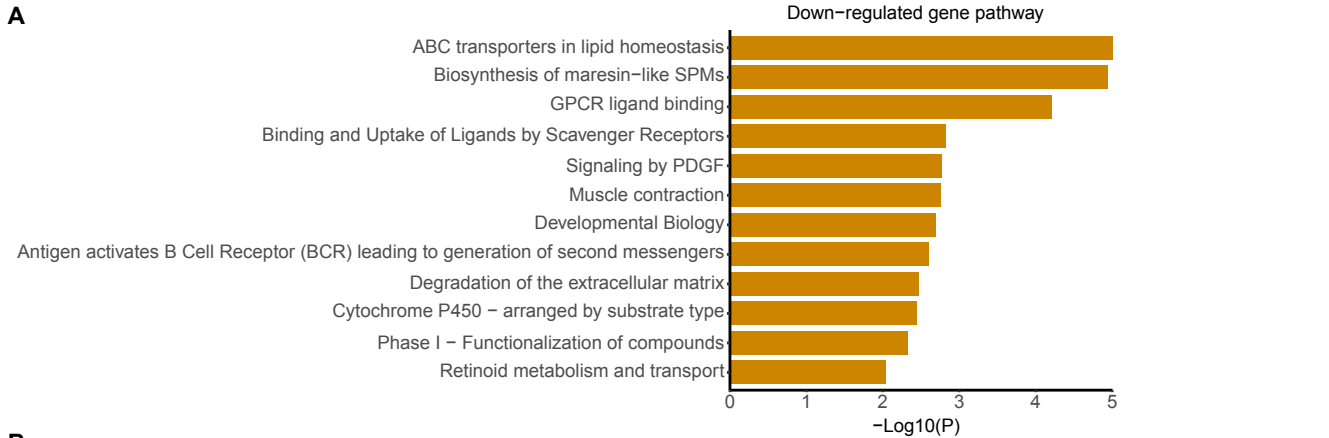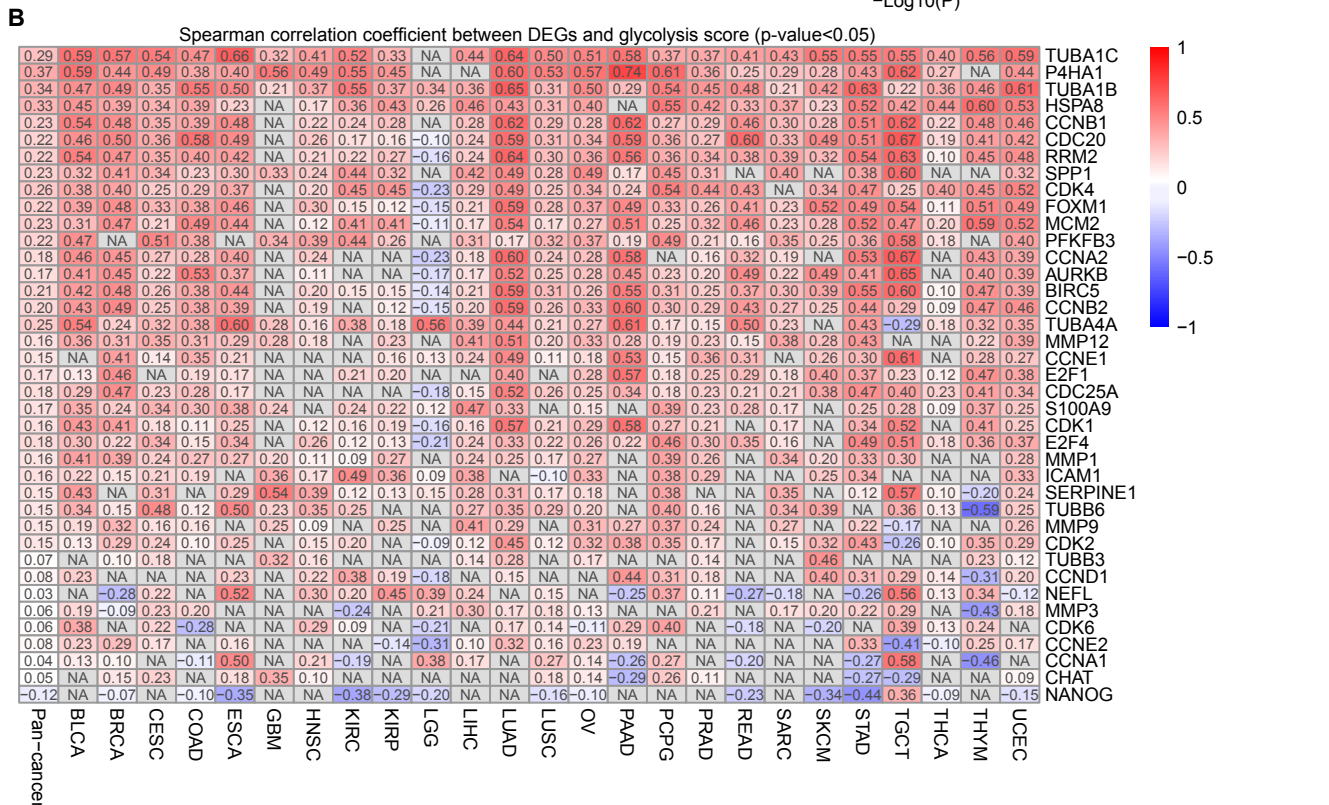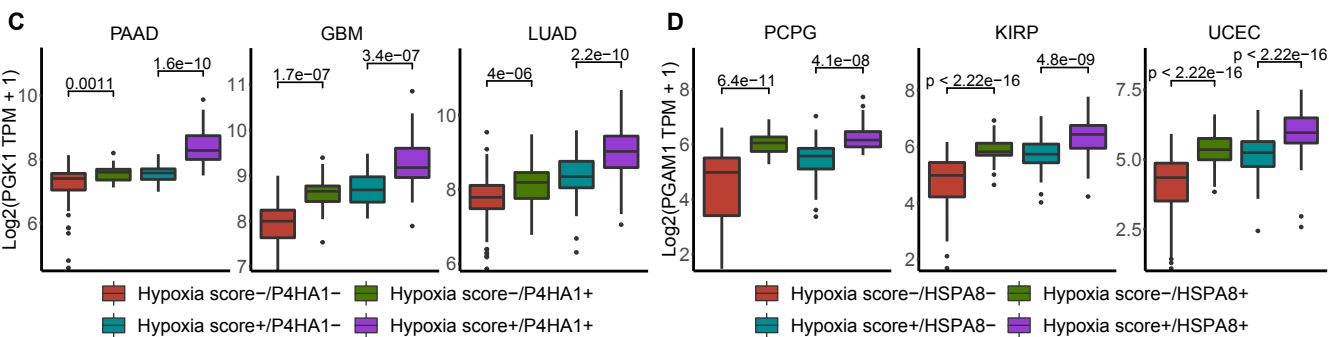

Supplement: Supplementary file 1 [file cancers-12-01788-s001.zip › Supplementary Files/Supplementary Figures/SF7.pdf]
